# Standards of Conditions During Preparations for the Summer Paralympic Games Between 2004 and 2012 Assessed by Polish Athletes

**DOI:** 10.1515/hukin-2015-0097

**Published:** 2015-01-12

**Authors:** Joanna Sobiecka, Wojciech Gawroński, Marta Kądziołka, Paweł Kruszelnicki, Jadwiga Kłodecka-Różalska, Ryszard Plinta

**Affiliations:** 1 Faculty of Motor Rehabilitation, University School of Physical Education, Krakow, Poland; 2 Department of Internal Medicine and Gerontology, Medical College, Jagiellonian Universit, Krakow, Poland; 3 Department of University School Informatization, University School of Physical Education, Krakow, Poland; 4 Department of Psychology, Institute of Sport, Warszawa, Poland; 5 School of Health Sciences in Katowice, Medical University of Silesia, Katowice, Department of Adapted Physical Activity and Sport, Chair of Physiotherapy

**Keywords:** disabled sports, preparation for competition, paralympians

## Abstract

The quality of training conditions affects sporting success, injuries and health. The aim of the work was to present the conditions during the preparations of Polish athletes for the Summer Paralympic Games 2004–2012. The study encompassed 271 paralympians: Athens (91), Beijing (89) and London (91), competing in 13 disciplines. The research was based on a two-part questionnaire by Kłodecka-Różalska adjusted for disabled sports, and was conducted one month before each PG. Part 1 contained 20 closed-ended questions regarding conditions during preparations, while Part 2 concerned socio-demographic and sports-related data. Three levels of conditions: good, satisfactory and poor, were identified. The analysis showed that while the relationships between the athletes were good in all the preparatory periods, the co-operation with the paralympic coaches worsened. The standards of accommodation, food and sports facilities lowered. Personal orthopaedic supply was satisfactory in London; personal sporting equipment was good at all PG. The quality of medical care was the highest in London. The co-operation with physicians, physiotherapists and massage therapists was satisfactory. Consultations with the dietician were sporadic and assessed as poor. Psychological consultations were rare but satisfactory in Beijing and London. Contacts with the mass media were poor at all PG. Although combining private life, work, and education with sport was satisfactory, it was increasingly difficult to manage, particularly before London. The conditions during preparations for the PG 2004–2012 varied. Improvement was noticed only in the quality of medical care and personal orthopaedic supply.

## Introduction

Polish athletes have been successfully competing in the Summer Paralympic Games (PG) since 1972. In the first PG in Heidelberg in 1972, Polish paralympians won 33 medals, thus placing Poland 6th out of 23 national team. In the last PG in London in 2012, the Polish paralympians won 36 medals and placed 9th out of 164 countries (Sobiecka, 2013).

According to many researchers, paralympic success is conditioned not only by the athletes’ own intensive work, the work of their coaches (Wu and Williams, 2001) or intensive training programmes (Tasiemski et al., 2004), but also by the standard of conditions provided during preparations for the PG.

The conditions concern various areas such as sports facilities (Blauwet and Willick, 2012; Jaarsma et al., 2014; Tasiemski et al., 2004), equipment (Rimmer et al., 2004), personal sporting equipment (Wu and Williams, 2001), and personal orthopaedic supply (Burkett, 2010; Rice et al., 2011).

According to Jaarsma et al. (2014), Rimmer et al. (2004), Tasiemski et al. (2004), and Webborn (2013), it is important to ensure appropriate food, accommodation and transportation for the athletes during training camps and national consultations.

Interpersonal relationships such as cooperation between the athletes and the paralympic coaches (Hanson and Nabavi, 2001; Sobiecka et al., 2011), cooperation among the athletes (Shapiro and Martin, 2010; Wu and Williams, 2001), and the athletes’ contacts with the media (Bruce, 2014; Chang et al., 2011; Webborn, 2013) are also crucial.

Another important factor determining the athletes’ preparation is the possibility to combine sports with other aspects of life, such as private life (Hanson and Nabavi, 2001; Jaarsma et al., 2014), education (Hanson and Nabavi, 2001; Sobiecka, 2013), and work (Hanson and Nabavi, 2001; Sobiecka, 2013; Tasiemski et al., 2004).

Furthermore, according to Gawroński and Sobiecka (2013) as well as Gawroński et al. (2013), the training process of athletes with disabilities should take place under the supervision of a medical team led by a medical specialist. According to Melion and Walsh (1999), the team should have clinical and scientific backup of medical consultants, a physiologist, a physiotherapist, a dietician, a psychologist, and a biomechanical engineer, responsible for preventing paralympians from injuries and health loss during preparations and competition.

Nonetheless, the above-listed individual suggestions were never a subject of a comprehensive study into the conditions in which the athletes of national teams prepare for the PG. In Poland, interest in this regard was first reported in 2004 (Sobiecka, 2005). The first study included athletes training for the Athens PG, and was then continued to include the subsequent PG. The aim of the present study was to provide a comprehensive assessment of the conditions in which Polish athletes trained for the PG between 2004 and 2012.

## Material and Methods

### Material

The study encompassed 271 athletes preparing for the PG in: Athens 2004*,* Beijing 2008 and London 2012. In 2004, the study participants (91 athletes) constituted 87.5% of the Polish National team, in 2008 (89 athletes) – 97.8%, while in 2012 (91 athletes) – 100%. For each PG, the athletes received nominations in 11 paralympic disciplines. The athletes were characterised by vision impairment or motor impairment. Paralympians with intellectual impairment were not included in the study (4 athletes in Athens and 9 athletes in London). Detailed characteristics of paralympians preparing for each PG are presented in Table 1.

### Methods

The study was conducted in four stages.

#### Stage I

Before each PG, the documentation of the Polish Paralympic Committee was studied. Next, a list of all the athletes qualified to participate in the PG in Athens 2004, Beijing 2008 and London 2012 was made. A database of Polish paralympians was created and used in the study (Sobiecka, 2013).

#### Stage II

Initial contacts were made with the authorities of sports clubs where the paralympians trained, paralympic coaches of various disciplines, and the athletes themselves. Next, one-to-one interviews took place during which the athletes were informed about the aim of the research and were asked for consent to participate in the study. The study was initiated after obtaining consent from all the abovementioned subjects.

#### Stage III

The study was conducted in Poland during central training camps and consultations, approx. four weeks prior to leaving for the PG in 2004, 2008 and 2012. Each time the study participants (athletes only, no coaches) were given detailed instructions.

The study was based on a two-part questionnaire by Kłodecka-Różalska adapted, with the author’s permission, for disabled sports and entitled “A survey of male and female athletes undergoing paralympic training”. Part 1 of the survey contained 20 closed-ended questions in which the respondents were asked to assess the conditions during preparations for the PG on a scale from 1 to 5 (5 – excellent, 4 – good, 3 – average, 2 – low, 1 – very negative). Part 2 concerned socio-demographic and sports-related data (Sobiecka et al., 2012).

#### Stage IV

The respondents’ answers were categorised and presented as percentages for each category of the assessment level (scale 1 to 5). Next, arithmetic means were calculated from the total of individual assessments. Based on the averaged assessments, three levels of conditions were identified: good (5.0-4.1), satisfactory (4.0-3.0), and poor (2.9-1.0). For each averaged opinion, the consistency of assessment of individual respondents was verified.

The opinions were considered significantly consistent if the confidence interval (CI) for the mean was <0.5 (alpha=0.05). Next, for each criterion the chi-squared test was performed, grouping assessment 5 and 4 (Group I) and assessment 3, 2 and 1 (Group II). If the chi-squared exceeded the cut-off point (resulting from chi-squared distribution for alpha=0.05 and df=2), the probability level was p<0.05.

The test was also performed for criteria that did not exceed the cut-off values, but only to compare the relationship between the opinions of respondents from Beijing 2008 with those from London 2012 (df=1). Those results were marked with a comment “*only for the Beijing – London relationship*”. The interpretation of the results shows significant differences in the assessment of selected criteria between the three (and additionally two) PG.

All stages of the study were performed following the code of ethics included in the International Ethical Guidelines on Biomedical Research involving Human Subjects published by the Council for International Organisations of Medical Sciences (CIOMS) together with the World Health Organisation (WHO), approved in 1982 and amended in 1993 and 2002 (International ethical guidelines for biomedical research involving human subjects. Geneva: Council for International Organizations of Medical Sciences, 2002 (access 13.06.2015)). Moreover, prior to initiating the study, the research protocol was approved by the Bioethics Committee at the District Chamber of Physicians in Cracow in 2012 (No 74 /KBL/OIL/2012 of 6th July, 2012).

## Results

Polish athletes participating in paralympic training were asked to comment on the conditions in which they prepared for the summer PG between 2004 and 2012. The assessed conditions included sports facilities, sports equipment, personal sports equipment, personal orthopaedic supply, transportation, accommodation during national training camps and consultations, interpersonal relationships, medical care, psychological care, physiological care, contacts with the media, and the ability to combine sports with other areas of the athletes’ life.

The analysis of the data presented in Table 2 showed that over the subsequent years of paralympic preparations, the quality of sporting facilities and personal sporting equipment was significantly satisfactory. The standard was the lowest according to the athletes who participated in the London PG, and the highest according to the athletes who participated in the Beijing PG (chi-squared test, p=0.002). The sporting equipment used by athletes during national training camps and consultations was significantly good only among athletes from the Beijing PG. With regard to personal orthopaedic supply, the low mean assessment reflects significant dissatisfaction among athletes from Athens and Beijing (chi-squared test, p=0.007). Significantly satisfactory personal orthopaedic supply was provided only before the London PG.

Most athletes assessed positively the standard of transportation to and from the training camps and sports consultations (Table 3). Although the athletes’ assessment was significantly satisfactory before all PG, its level was the highest before Beijing (Beijing vs. London chi-squared test, p=0.044). Significant lowering of the standard of accommodation, from good to satisfactory (chi-squared test, p=0.044), and of food (chi-squared test, p=0.028) was observed over the subsequent years, and particularly from 2008 to 2012.

Despite the change in cooperation with paralympic team coaches, from good to satisfactory, interpersonal relationships were assessed significantly high in all the study periods (Table 4).

The cooperation between the athletes themselves and the atmosphere during training camps and consultations before each PG were assessed very high.

Medical conditions were evaluated the highest by the athletes preparing for the London PG (Table 5). Medical care understood as cooperation with the physician, physiotherapist, massage therapist as well as wellness facilities provided during preparations for the PG were significantly satisfactory. Between 2004 and 2012, the athletes’ mean assessment of particular aspects of medical care systematically increased (chi-squared test, p=0.023), although not all athletes made use of it. Only a very small group of paralympians had consultations with a dietician and they were assessed negatively. Few paralympians sought psychological care, which they assessed significantly satisfactory before Beijing and London (chi-squared test, p<0.001). A physiologist was not present at any stage of the paralympic preparations.

The athletes’ contacts with the media that accompanied them during the preparations were significantly poor (Table 6).

The ability to combine sport with personal life, education and work was significantly satisfactory; however, it became increasingly difficult, especially before the London PG (Table 7).

## Discussion

The athletes’ opinions varied greatly and ranged from extremely positive to extremely negative. This confirms that between 2004 and 2012, not all areas of conditions for paralympic preparations were satisfactory. Our study is the first to present a comprehensive assessment of the conditions of paralympic preparations. To our knowledge, there are no similar studies in the literature, hence it is impossible to compare our results with those of other authors.

The standard of sports facilities, appropriately adapted to the functional needs of athletes with disabilities, plays a crucial role in paralympic preparation. According to Blauwet and Willick (2012), Jaarsma et al., (2014) and Tasiemski et al. (2004), such facilities enable athletes to move freely, giving them access to all areas. Whether a given facility meets the standards depends not only on the team coach, whose duty is to plan and select optimal training facilities (Sobiecka et al., 2012), but also on the economic possibilities of sports organisations responsible for covering the costs of paralympic preparations. In recent years, Polish paralympic sport has been negatively affected by the economic crisis, thus receiving insufficient funds from the national budget, and by the Ministry’s endeavours to generate extra savings (Sobiecka, 2013). This was reflected by the athletes’ opinions on the standard of sports facilities, which not only was not satisfactory but even worsened before London.

The same concerned sporting equipment and personal sporting equipment, especially before London. Vanlandewijck (2006) along with Wu and Williams (2001) claim that high quality sporting equipment not only facilitates the achievement of better paralympic results but, above all, ensures the disabled athletes’ safety. Laing and Carr (2005) observed that appropriate sports clothing was not only comfortable, but, more importantly, in case of a fall, it lessened the injury. In view of the above, the authorities of the Polish Paralympic Committee should not remain inert in the planning of the budget and the organisation of the preparations for the next PG.

The athletes’ opinions regarding the conditions of individual orthopaedic supply, particularly in 2004 and 2008, are alarming. Only before London, its level was satisfactory according to most paralympians. This is a good sign. Firstly, because the needs of Polish paralympians in this regard (Sobiecka, 2007) are finally beginning to be met thanks to sponsors and the National Fund for the Rehabilitation of People with Disabilities. Secondly, achieving increasingly good sporting results would not be possible without technologically advanced personal orthopaedic supply. This is particularly evident in the world’s paralympic sport, both with regard to adaptation/modification and structural evolution of the equipment to fit specific sports disciplines (Burkett, 2010; Rice et al., 2011).

National training camps and consultations are thought to be the core of paralympic sports training of the national team. Therefore, good standards of accommodation, food and transportation are very important (Jaarsma et al., 2014; Rimmer et al., 2004; Tasiemski et al., 2004; Webborn, 2013). During training camps, the athletes try to make the best use of their time for optimal preparation for the PG. Sadly, the standards of food, accommodation and transport during training camps and consultations lowered from good to satisfactory.

It is believed that sporting success is affected not only by appropriate training and the athletes’ sporting skills, but also, due to high stress levels, by good interpersonal relationships, including cooperation with the coaches and good atmosphere among the athletes themselves. The hypothesis of the impact of the coach-athlete relationship has been confirmed by many authors (Hanson and Nabavi, 2001; LaVoi, 2007; Smith and Smoll, 2007; Sobiecka et al., 2011). In our study, communication, which is a key factor for a successful relationship between coaches and athletes, was very good (Sobiecka, 2011). This may affect positively also the following paralympic preparations in 2016.

Our study also showed some positive changes that took place with regard to the quality of medical care and concerned the athletes’ cooperation with the physician, physiotherapist, massage therapist and the possibility to use wellness facilities. Although between 2004 and 2012, the assessment of medical care was far from good, the greatest changes took place before the London PG thanks to the chief of the Polish medical team.

According to Gawroński et al. (2013), in order to ensure safe training and competing conditions, it was necessary to impose the execution of systematic prophylactic medical check-ups for sports, completed with a physician’s certificate confirming the athletes’ health status. The introduction of simple rules of prophylaxis, such as obligatory multi-specialist sports medicine check-ups before the London PG, not only was met with the athletes’ approval but also contributed to lowering of the number of injuries and illnesses during the PG. It was also reflected by the athletes’ health status and better results (Poland placed 9th out of 161 national teams, Sobiecka, 2013). The study by Gawroński and Sobiecka (2013) confirmed the importance of prophylactic, multi-specialist check-ups conducted at the Main Sports Medicine Centre in Warsaw, or other designated multi-specialist centres. However, such check-ups should be performed not only just before the PG, but systematically, throughout the entire cycle of preparations in cooperation with a medical team experienced in dealing with people with disabilities and familiar with the specificity of disabled sports.

Emotional support is also a key factor in the process of paralympic preparations. Cummins and Kelly (2012) along with Porter (2003) claim that success at important competitions is not only the result of physical training but also of psychological preparation. Our data shows that the number of athletes who had access to a psychologist increased from the Beijing PG; most athletes considered it sufficient to prepare for the PG. Hopefully, before the next PG, access to psychological care will be provided to all national team representatives.

There is a general belief that a multifaceted cooperation between sports associations of athletes with disabilities and the media has a considerable impact on both increasing the public awareness regarding people with disabilities and popularising the idea of paralympic sport (Blauwet and Willick, 2012; Bruce, 2014; Tasiemski et al., 2004; Webborn, 2013). The London PG, during which the paralympians’ image was professionally promoted in the English media, are a good example of effective promotional actions. The paralympians were presented as people achieving sporting success and not as people who struggle with their disability (Webborn, 2013). Such actions lead to a positive perception of the value of sport, also in the aspect of the athletes’ quality of life. The Polish organisations for disabled sports, and especially the Polish Paralympic Committee, should have a similar objective, particularly in the light of the paralympians’ negative opinions regarding contacts with mass media at all stages of paralympic preparations.

According to Hanson and Nabavi (2001), Jaarsma et al. (2014), and Jones and Howe (2005), training schedules should necessarily take into consideration the athletes’ everyday life needs. Worryingly, in our study, the respondents’ comments varied greatly. Practicing sports, both by single athletes and athletes in a relationship, became increasingly difficult to combine with private life, especially before the London PG. Likely, a considerable group of athletes sacrificed their private life for paralympic preparations (Sobiecka, 2013).

According to Hanson and Nabavi, (2001), the ability to combine work with other areas of life is another key issue. The importance of work for the functioning of athletes with disabilities was reported by Sobiecka (2013) and Tasiemski et al. (2004). Beside social benefits, work gives financial independence, while the experience gained during sporting career can prove useful at work. A similar relationship was observed for education (Sobiecka, 2004). Kraemer and Fleck (2004) suggest that the process of education is affected not only by the individualisation of teaching and support from teachers and family, but also by the attitude of the sporting society, mainly the coaches of young athletes. Coaches should remember about this very important aspect during paralympic preparations. The majority of paralympians in our study had secondary and tertiary education; very few had primary or vocational education.

In the light of the above discussed results, it seems well-founded to suggest to the Polish Paralympic Committee that they perform a detailed analysis of the conditions in which the Polish paralympic athletes prepared for the PG between 2004 and 2012, to introduce important organisational and training changes to the benefit of athletes preparing for the summer PG in Rio de Janeiro.

## Conclusions

According to the Polish paralympians, the conditions in which they prepared for the Paralympic Games between 2004 and 2012 varied significantly, and the changes that took place were not always positive. The atmosphere at training camps and consultations as well as the cooperation among the athletes themselves, were the only aspects to be assessed high during all preparation periods. Over the three study periods, an improvement was observed only in medical care (cooperation with the physician, physiotherapist and massage therapist) and personal orthopaedic supply.

## Figures and Tables

**Table 1:** Detailed characteristics of the studied athletes preparing for the Paralympic Games between 2004 and 2012

Socio-demographic and sports characteristics	Athletes participating in paralympic training					
	Athens 2004		Beijing 2008		London 2012	
	n	%	n	%	n	%
SEX						
Women	30	33.0	31	34.8	30	33.0
Men	61	67.0	58	65.2	61	67.0
Total	91	100	89	100	91	100
AGE AT PARALYMPIC GAMES [years]						
mean	32.7		32.1		31.8	
SD	10.9		9.7		9.6	
x_min_ - x_max_	15–57		15–61		14–57	
MARITAL STATUS						
Single	46	50.5	51	57.4	52	57.1
Married	40	44.0	36	40.4	30	33.0
Divorced	5	5.5	0	0	6	6.6
Common-law partnership	0	0	2	2.2	3	3.3
EDUCATION						
Tertiary	23	25.2	23	25.9	29	31.8
Secondary	13	14.3	21	23.6	25	27.5
Secondary vocational	26	28.6	19	21.3	25	27.5
Basic vocational	17	18.7	19	21.3	8	8.8
Primary	12	13.2	7	7.9	4	4.4
WORK						
Professionally active – working	49	53.8	49	55.1	43	47.2
Professionally active – unemployed	3	3.3	5	5.6	6	6.6
Professionally passive – not working	17	18.7	13	14.6	17	18.7
Professionally passive – school and university students	22	24.2	22	24.7	25	27.5
DISABILITY TYPE						
Visual impairment	5	5.5	13	14.6	2	2.2
Motor impairment	86	94.5	76	85.4	89	97.8
YEARS OF COMPETING BEFORE RECEIVING PARALYMPIC NOMINATION [years]						
mean	10.4		12.3		12.1	
SD	7.5		6.0		6.1	
x_min_ - x_max_	2–43		2–27		2–35	
NUMBER OF PARALYMPIC GAMES ATTENDED						
One	49	53.8	38	42.7	38	41.8
Multiple	42	46.2	51	57.3	53	58.2

**Table 2:** Detailed analysis of sports facilities, equipment, personal equipment and personal orthopaedic supply used by the athletes during paralympic preparations (athletes’ assessment)

Study participants		Assessment categories						Mean assessment	Category of conditions*

Year of the PG	n	5	4	3	2	1	n/a		

				[%]			[%]		
**SPORTS FACILITIES**									
*chi-2 test: 12.99; p = 0.002*									
2004	91	30.8	47.2	14.3	5.5	2.2	0	4.0	Satisfactory*
2008	89	25.8	50.6	18.0	4.5	1.1	0	4.0	Satisfactory*
2012	91	15.4	40.6	36.3	4.4	3.3	0	3.6	Satisfactory*
**SPORTING EQUIPMENT**									
2004	91	29.7	40.6	18.7	7.7	3.3	0	3.9	Satisfactory*
2008	89	39.3	37.1	16.9	6.7	0	0	4.1	Good*
2012	91	24.2	39.6	26.3	5.5	4.4	0	3.7	Satisfactory*
**PERSONAL SPORTING EQUIPMENT**									
*chi-2 test: 6.25; p = 0.044*									
2004	91	16.5	41.7	23.1	13.2	5.5	0	3.5	Satisfactory*
2008	89	30.3	41.6	18.0	10.1	0	0	3.9	Satisfactory*
2012	91	15.4	33.0	19.7	17.6	3.3	11.0	3.4	Satisfactory*
**PERSONAL ORTHOPAEDIC SUPPLY**									
*chi-2 test: 9.80; p = 0.007*									
2004	91	3.3	26.4	13.2	13.2	23.1	20.8	2.6	Poor*
2008	89	10.1	10.1	12.4	11.2	22.5	33.7	2.7	Poor*
2012	91	11.0	17.6	5.5	6.6	6.6	52.7	3.3	Satisfactory*

* level of statistical significance: p<0.05

**Table 3:** Detailed analysis of transport, accommodation and food during national training camps and consultations during paralympic preparations (athletes’ assessment)

Study participants		Assessment categories						Mean assessment	Category of conditions*

Year of the PG	n	5	4	3	2	1	n/a		

				[%]			[%]		
**TRANSPORT TO NATIONAL TRAINING CAMPS AND CONSULTATIONS**									
*chi-2 test: 4.1; df = 1; p = 0.044 (only for Beijing vs. London)*									
2004	91	23.1	36.2	22.0	9.9	8.8	0	3.5	Satisfactory*
2008	89	28.1	37.0	18.0	12.4	0	4.5	3.8	Satisfactory*
2012	91	16.5	36.3	27.5	12.0	6.6	1.1	3.4	Satisfactory*
**ACCOMMODATION DURING NATIONAL TRAINING CAMPS AND CONSULTATIONS**									
*chi-2 test: 10.39; p = 0.006*									
2004	91	37.4	47.2	11.0	0	3.3	1.1	4.2	Good*
2008	89	32.6	42.7	18.0	2.2	0	4.5	4.1	Good*
2012	91	18.7	46.1	29.7	3.3	1.1	1.1	3.8	Satisfactory*
**FOOD DURING NATIONAL TRAINING CAMPS AND CONSULTATIONS**									
*chi-2 test: 4.8; df = 1; p = 0.028 (only for Beijing vs. London)*									
2004	91	40.6	42.9	13.2	1.1	1.1	1.1	4.2	Good*
2008	89	44.9	36.0	10.1	1.1	3.4	4.5	4.2	Good*
2012	91	23.1	48.3	17.6	6.6	3.3	1.1	3.8	Satisfactory*

* level of statistical significance: p<0.05

**Table 4:** Detailed analysis of cooperation between the athletes and the paralympic team coaches; atmosphere and relationships among athletes preparing for the Paralympic Games (athletes’ assessment)

Study participants		Assessment categories					Mean assessment	Category of conditions*
Year of the PG	n	5	4	3	2	1		

				[%]				
**COOPERATION WITH PARALYMPIC TEAM COACHES**								
2004	91	53.8	28.6	7.7	5.5	4.4	4.2	Good*
2008	89	52.8	20.2	14.6	5.6	6.8	4.1	Good*
2012	91	41.8	30.9	12.0	12.0	3.3	4,0	Satisfactory*
**ATMOSPHERE AND RELATIONSHIPS AMONG ATHLETES**								
2004	91	46.2	36.2	7.7	6.6	3.3	4.2	Good*
2008	89	38.2	47.2	10.2	2.2	2.2	4.2	Good*
2012	91	46.1	33.0	13.2	5.5	2.2	4.2	Good*

* level of statistical significance: p<0.05

**Table 5:** Detailed analysis of medical, psychological and physiological care provided to athletes preparing for the Paralympic Games (athletes’ assessment)

Study participants		Assessment categories						Mean assessment	Category of conditions*

Year of the PG	n	5	4	3	2	1	Lack of care and cooper. [%]		

				[%]					
**MEDICAL CARE**									
*chi-2 test: 5,2; df = 1; p = 0.023 (only for Beijing vs. London)*									
2004	91	6.6	16.5	19.7	15.4	33.0	8.8	2.4	Poor*
2008	89	13.5	11.2	14.6	21.4	39.3	0	2.4	Poor*
2012	91	5.5	19.8	17.6	5.5	12.0	39.6	3.0	Satisfactory*
**WELLNESS AFTER TRAINING AND COMPETITIONS**									
2004	91	22.0	28.5	22.0	13.2	13.2	1.1	3.3	Satisfactory*
2008	89	18.0	22.5	25.8	15.7	18.0	0	3.1	Satisfactory*
2012	91	14.3	33.0	18.6	11.0	8.8	14.3	3.4	Satisfactory*
**COOPERATION WITH THE PHYSIOTHERAPIST**									
2004	91	13.2	16.5	6.6	7.7	22.0	34.0	2.9	Poor
2008	89	14.6	13.5	9.0	9.0	10.1	43.8	3.2	Satisfactory*
2012	91	23.1	23.1	18.6	7.7	4.4	23.1	3.7	Satisfactory*
**COOPERATION WITH THE MASSAGE THERAPIST**									
*chi-2 test: f = 1; p = 0.046 (only Beijing vs. London)*									
2004	91	28.6	25.2	9.9	3.3	15.4	17.6	3.5	Satisfactory*
2008	89	24.7	15.7	10.1	14.6	9.0	25.9	3.6	Satisfactory*
2012	91	26.4	34.1	18.6	3.3	3.3	14.3	3.9	Satisfactory*
**COOPERATION WITH THE DIETICIAN**									
2004	91	8.8	7.7	7.7	11.0	25.3	39.5	2.4	Poor*
2008	89	3,4	2.2	4.5	5.6	12.4	71.9	2.2	Poor*
2012	91	2.2	2.2	1.1	3.3	6.6	84.6	2.4	Poor*
**COOPERATION WITH THE PSYCHOLOGIST**									
*chi-2 test: 25.1; p < 0.001*									
2004	91	5.5	2.2	3.3	8.8	34.1	46.1	1.8	Poor*
2008	89	3.4	4.5	0	2.2	1.1	88.8	3.6	Satisfactory*
2012	91	8.8	16.5	5.5	3.3	6.6	59.3	3.4	Satisfactory*
**COOPERATION WITH THE PHYSIOLOGIST**									
2004	91	0	0	0	0	0	100	0	LC
2008	89	0	0	0	0	0	100	0	LC
2012	91	0	0	0	0	0	100	0	LC

* level of statistical significance: p<0.05; LC- Lack of cooperation

**Table 6:** Detailed analysis of the athletes’ contacts with the mass media during paralympic preparations (athletes’ assessment)

Study participants		Assessment categories						Mean assessment	Category of conditions*

Year of the PG	n	5	4	3	2	1	Lack of contacts [%]		

				[%]					
**CONTACTS WITH MASS MEDIA**									
2004	91	5.5	12.0	26.4	23.2	20.9	12.0	2.5	Poor*
2008	89	9.0	9.0	21.4	26.9	19.1	14.6	2.6	Poor*
2012	91	3.3	6.6	19.7	20.9	17.6	31.9	2.4	Poor*

* level of statistical significance: p<0.05

**Table 7:** Detailed analysis of the possibility to combine private life, work and education with sport by athletes preparing for the Paralympic Games (athletes’ assessment)

Study participants		Assessment categories						Mean assessment	Category of conditions*

Year of the PG	n	5	4	3	2	1	n/a		

				[%]			[%]		
**POSSIBILITY TO COMBINE PRIVATE LIFE AND SPORT**									
2004	91	23.2	40.4	23.2	11.0	2.2	0	3.7	Satisfactory*
2008	89	19.1	40.5	33.7	2.2	4.5	0	3.7	Satisfactory*
2012	91	8.8	43.9	35.2	8.8	3.3	0	3.5	Satisfactory*
**POSSIBILITY TO COMBINE WORK AND SPORT**									
2004	91	15.3	23.2	33.0	13.2	3.3	12.0	3.4	Satisfactory*
2008	89	11.2	20.2	21.4	5.6	6.7	34.8	3.4	Satisfactory*
2012	91	14.3	7.7	17.6	16.5	5.5	38.4	3.1	Satisfactory*
**POSSIBILITY TO COMBINE EDUCATION AND SPORT**									
2004	91	17.6	25.3	19.8	4.4	3.3	29.6	3.7	Satisfactory*
2008	89	9.0	14.6	23.6	5.6	4.5	42.7	3.3	Satisfactory*
2012	91	3.3	9.9	9.9	4.4	2.2	70.3	3.3	Satisfactory*

* level of statistical significance: p<0.05

## References

[b1-jhk-48-111] Blauwet C, Willick SE (2012). The Paralympic Movement: using sports to promote health, disability rights, and social integration for athletes with disabilities. PM&R.

[b2-jhk-48-111] Brukett B (2010). Technology in Paralympic sport: performance enhancement or essential for performance?. Br J Sports Med.

[b3-jhk-48-111] Bruce T (2014). Us and them: the influence of discourses of nationalism on media coverage of the Paralympic. Disability & Society.

[b4-jhk-48-111] Chang IY, Crossman J, Taylor J, Walker D (2011). One World, one dream: a qualitative comparison of the newspaper coverage of the 2008 Olympic and Paralympic Games. Int J Sport Commun.

[b5-jhk-48-111] Cummins P, Kelly C (2012). Caring and sharing: continuity of care in the pursuit of excellence. Sport Exerc Psychol.

[b6-jhk-48-111] Gawroński W, Sobiecka J (2013). Medical care and sports medical examinations in disabled athletes before the 2008 and 2012 Summer Paralympic Games. Med Sport Pract.

[b7-jhk-48-111] Gawroński W, Sobiecka J, Malesza J (2013). Fit and healthy Paralympians – medical care guidelines for disabled athletes: a study of the injuries and illnesses incurred by the Polish Paralympic team in Beijing 2008 and London 2012. Br J Sports Med.

[b8-jhk-48-111] Hanson C, Nabavi D (2001). The effects of sports on level of community integration as reported by persons with spinal cord injury. Am J Occup Ther.

[b9-jhk-48-111] Jaarsma EA, Geertzen JH, de Jong R, Dijkstra PU, Dekker R (2014). Barriers and facilitators of sports in Dutch Paralympic athletes: An explorative study. Scand J Med Sci Spor.

[b10-jhk-48-111] Jones C, Howe PD (2005). The conceptual boundaries of sport for the disabled: classification and athletic performance. J Philos Sport.

[b11-jhk-48-111] Kraemer WJ, Fleck SJ (2004). Strength Training for Young Athletes. Creating individualized programs.

[b12-jhk-48-111] Laing RM, Carr DJ, Shiho R (2005). Textiles in Sport. Is protection part of the game? Protection against impact using clothing and personal equipment.

[b13-jhk-48-111] LaVoi NM (2007). Expanding the interpersonal dimension: closeness in the coach-athlete relationship. Int J Sports Sci Coach.

[b14-jhk-48-111] Melion MB, Walsh WM, Melion MB (1999). Sports Medicine Secrets. The team physician.

[b15-jhk-48-111] Porter K (2003). The mental athlete: Inner training for peak performance in all sports. Mental training for specific needs.

[b16-jhk-48-111] Rice I, Hettinga FJ, Laferrier J, Sporner ML, Heiner CM, Brukett B, Vanlandewijck YC, Thompson WR (2011). The Paralympic athlete: Handbook of sports medicine and science. Biomechanics.

[b17-jhk-48-111] Rimmer JH, Riley B, Wang E, Rauworth A, Jurkowski J (2004). Physical activity participation among persons with disabilities: barriers and facilitators. Am J Prev Med.

[b18-jhk-48-111] Shapiro DR, Martin JJ (2010). Athletic identity, affect and peer relations in youth athletes with physical disabilities. Disabil Health J.

[b19-jhk-48-111] Smith RE, Smoll FL, Smith D, Bar-Eli M (2007). Behavioral research and intervention in youth sports. Essential readings in sport and exercise psychology.

[b20-jhk-48-111] Sobiecka J (2004). Education, socio-occupational structure and occupational work of Polish athletes participating in Paralympic Games Sydney 2000. Eukrasia.

[b21-jhk-48-111] Sobiecka J (2005). Some selected aspects concerning the preparation of the Polish national team members for the Summer Paralympic Games held in Athens in 2004. Med Sport.

[b22-jhk-48-111] Sobiecka J (2007). Barriers hindering Paralympic sports of disabled competitors. Pol J Environ Stud.

[b23-jhk-48-111] Sobiecka J (2013). The image of a Polish Paralympian.

[b24-jhk-48-111] Sobiecka J, Plinta R, Cichoń K, Drobniewicz K (2011). The relations that occurred between the athletes and coaches of the national team during their preparations for the Paralympic Games (according to the athletes). J Orthop Trauma Surg Relat Res.

[b25-jhk-48-111] Sobiecka J, Plinta R, Drobniewicz K, Kłodecka-Różalska J, Cichoń K (2012). Conditions for preparations for the 2008 Beijing Paralympic Games in the opinion of the Polish national team. Biomed Hum Kinet.

[b26-jhk-48-111] Tasiemski T, Kennedy P, Gardner BP, Blaikley RA (2004). Athletic identity and sports participation in people with spinal cord injury. Adapt Phys Act Q.

[b27-jhk-48-111] Vanlandewijck Y (2006). Sport science in the Paralympic movement. J Rehabil Res Dev.

[b28-jhk-48-111] Webborn N (2013). London 2012 Paralympic Games: bringing sight to the blind?. Br J Sports Med.

[b29-jhk-48-111] Wu SK, Williams T (2001). Factors influencing sport participation among athletes with spinal cord injury. Med Sci Sport Exer.

